# Dendritic Cells and Multiple Sclerosis: Disease, Tolerance and Therapy

**DOI:** 10.3390/ijms14010547

**Published:** 2012-12-27

**Authors:** Mohammad G. Mohammad, Masoud Hassanpour, Vicky W. W. Tsai, Hui Li, Marc J. Ruitenberg, David R. Booth, Jordi Serrats, Prue H. Hart, Geoffrey P. Symonds, Paul E. Sawchenko, Samuel N. Breit, David A. Brown

**Affiliations:** 1 The Laboratory of Neuroinflammation, St Vincent’s Centre for Applied Medical Research, University of New South Wales, Sydney 2010, Australia; E-Mails: m.mohammad@amr.org.au (M.G.M.); m.hassanpour@amr.org.au (M.H.); v.tsai@amr.org.au (V.W.W.T.); h.li@amr.org.au (H.L.); g.symonds@amr.org.au (G.P.S.); s.breit@amr.org.au (S.N.B.); 2 School of Biomedical sciences, The University of Queensland, Brisbane 4072, Australia; E-Mail: m.ruitenberg@uq.edu.au; 3 Institute for Immunology and Allergy Research, Westmead Millennium Institute, University of Sydney, Sydney 2145, Australia; E-Mail: david_booth@wmi.usyd.edu.au; 4 Jordi Serrats: Neuroinflammation Disease Biology Unit, Lundbeck Research USA, 215 College Road, Paramus, NJ 07652, USA; E-Mail: jsrt@lundbeck.com; 5 Telethon Institute for Child Health Research, Centre for Child Health Research, University of Western Australia, Perth 6872, Australia; E-Mail: prueh@ichr.uwa.edu.au; 6 Laboratory of Neuronal Structure and Function, The Salk Institute for Biological Studies, 10010 North Torrey Pines Road, La Jolla, CA 92037, USA; E-Mail: sawchenko@salk.edu

**Keywords:** multiple sclerosis, dendritic cell, experimental autoimmune encephalomyelitis, cervical lymph node, molecular therapy, cellular therapy, site directed local therapy

## Abstract

Multiple sclerosis (MS) is a devastating neurological disease that predominantly affects young adults resulting in severe personal and economic impact. The majority of therapies for this disease were developed in, or are beneficial in experimental autoimmune encephalomyelitis (EAE), the animal model of MS. While known to target adaptive anti-CNS immune responses, they also target, the innate immune arm. This mini-review focuses on the role of dendritic cells (DCs), the professional antigen presenting cells of the innate immune system. The evidence for a role for DCs in the appropriate regulation of anti-CNS autoimmune responses and their role in MS disease susceptibility and possible therapeutic utility are discussed. Additionally, the current controversy regarding the evidence for the presence of functional DCs in the normal CNS is reviewed. Furthermore, the role of CNS DCs and potential routes of their intercourse between the CNS and cervical lymph nodes are considered. Finally, the future role that this nexus between the CNS and the cervical lymph nodes might play in site directed molecular and cellular therapy for MS is outlined.

## 1. Introduction

Multiple sclerosis (MS) is a chronic demyelinating inflammatory disease of the central nervous system (CNS) [1,2]. It has a number of clinical presentations that fall into two broad categories: relapsing remitting-disease and progressive disease [1,2]. Many patients that present with relapsing-remitting disease evolve into a relentlessly escalating form known as secondary progressive MS [1,2]. While the triggering factors in disease are hotly debated, perhaps because they are in themselves heterogeneous, the key role of the immune system is well established [3]. One of the strongest genetic associations with MS disease is a polymorphism of the human leukocyte antigen complex (HLA-DRB1*1501), which is intimately involved in antigen presentation, and several other HLA region variants are also strongly associated with MS [4–10]. Further, single nucleotide polymorphisms (SNPs) in co-receptors essential for effective antigen presentation have also been identified using genome-wide association studies (GWAS) [5]. The essential role played by antigen-presenting cells, like dendritic cells (DCs), in MS progression is perhaps best exemplified by the finding that many approved therapies, as well as new treatments in late phase clinical trials such as BG12, lead to significant modification of DCs [11–16].

A number of innate and adaptive immune cells are implicated in the establishment and progression of MS and its dominant animal model, experimental autoimmune encephalomyelitis (EAE). Aside from DCs [17], and their important role in T-cell polarization during EAE [17], the immune cells that are thought to have a role in EAE include gamma-delta-T-cells [18]; NKT-cells and NK cells [19–23]; B-cells [24–26]; mast cells [27–29]; macrophages (reviewed in [30]); and microglia (reviewed in [31,32]). Additionally, astrocytes appear to participate as a quasi-innate immune cell by virtue of their capacity to present antigen to T-lineage cells [33–39], a capacity shared with B-cells [40,41].

T-lymphocytes are a key mediator of disease activity [3], though the number of circulating CNS-reactive T-cells present in MS patients and normal subjects is similar [42]. Thus, the presence of CNS antigen reactive T-cells alone, is insufficient to induce disease and other important factors must be involved. As antigen presentation, most commonly by DCs, is essential for most T-cell responses [43] it seems reasonable to assume that regulation by this cell type will provide important checks and balances for T-cell activation. For this reason, we focus this mini-review on the role of DCs in immune surveillance in health and in modulating CNS-specific immune responses in disease. A more extensive review on other actions of DCs was recently published by Colton *et al*. [44] In this context the first question that needs to be answered is whether these mechanisms are required or likely to be present.

## 2. DCs in Health and Disease

Dendritic cells (DCs) are professional antigen presenting cells that link the innate and adaptive immune systems [43]. They have a key role in activating, shaping, and in some cases preventing damaging anti-CNS immune responses that are characteristic of MS [45–49]. To undertake these functions (outside the CNS, at least), DCs patrol mucosal surfaces and solid organs and respond to pathogenic challenges by engulfing antigen, processing it, and then presenting it to lymphocytes at the site of the insult or in the regional lymph nodes [50]. To regulate systemic immunity, after antigen challenge, some DCs migrate to draining lymph nodes [50]. Here, they not only activate effector lymphocytes, but also play an important role in the control of inappropriate immune responses [17,50,51].

## 3. MS, Vitamin D and DC Activity

Environmental factors are likely to play a major role in MS. This is best highlighted by studies in identical twins, in whom the concordance rate for the development of MS is only about 30% [52]. One environmental factor may well be sun exposure, as the global incidence of MS is positively related to latitude. While vitamin D levels and exposure to ultraviolet light are intimately related, both latitude and vitamin D levels are independently associated with the risk of MS [53–55].

Administration of vitamin D modulates disease severity in animal models of MS and might increase disease free periods by reducing relapse frequency in humans [55]. Vitamin D is well known to affect a number of immune cells (reviewed in [56]) and is intimately involved in the regulation of DC function, with increased concentrations of vitamin D leading to the induction of immune regulatory actions. These include the up regulation of TGF-β and IL-10 [57], which have been shown to increase the production of T-regulatory cells, [17] whose actions are broadly disease-suppressive. Further, in a recent GWAS study, a polymorphism in the gene block containing the *Cyp27B1* gene that encodes the enzyme Cyp27B1 hydroxylase which catalyses the conversion of 1-hydroxy-vitamin D to its biologically active form, 1,25-hydroxy-vitamin D, was found to be associated with MS [5]. It is thus possible that the lack of the Cyp27B1 substrate, 25-hydroxy-vitamin D, resulting from reduced exposure to UV light, leads to reduced active 1, 25-hydroxy-vitamin D, and, as a consequence, reduces the ability of DCs to down-regulate harmful immune responses [56].

## 4. Immunoregulatory DCs

DCs are key cells in immune regulation [58–62]. Their presentation of antigen without appropriate costimulatory molecule expression leads to T-cell death/anergy or induction of the T-regulatory phenotype [63]. Activation with appropriate costimulation leads to the ability to induce the full range of T-helper-cell phenotypes, including the regulatory phenotypes [58–62]. It has long been recognized that thymic DCs are important for establishment of central tolerance, by eliminating highly autoreactive T-cells [64]. However, more recently, it has been appreciated that DCs also participate in the maintenance of peripheral tolerance [65–67]. Whilst a tolerogenic phenotype is helpful in preventing or resolving diseases like MS, in some circumstance, it may be harmful. For example, tumors down-regulate anti-tumor immune responses by secreting factors that favor the development of DCs that induce T-regulatory cell differentiation [68]. These T-regulatory cells then actively reduce anti-tumor immunity, allowing the tumor to evade the systemic immune response [69]. A less sinister example is the acceptance of human liver allografts without immunosuppression. Here it is thought that DCs might similarly promote T-regulatory function in lymph nodes draining the liver [70–72].

Critical appraisal of new data on DC function, along with a large body of literature in experimental animals, would suggest that defects in DC development might lead to impaired tolerance and immune activation. Genetic mutations leading to developmental defects in DCs have recently been described in humans [73,74]. These mutations lead not only to impaired resistance to infection, but also to an increase in autoinflammatory disease associated with aberrant DC development [73–75]. For example, multiple SNPs in interferon response factor-8 (IRF8) have been associated with human DC developmental defects [73]. When this gene is knocked out in mice there is an absence of multiple subsets of DCs, some of which play a significant role in immunoregulation by producing type 1 interferons, cytokines that are used in MS therapy [76,77]. Also, a genetic polymorphism in the IRF8 gene is a highly significant predisposing factor to MS [5,78,79]. Finally, loci such as *MLANA*, *EOMES* and *TNFRSF1A* are now known to be associated with MS with their pathways predominantly expressed in DCs [4]. Taken together, these findings provide a strong rationale for positing an involvement of DCs in the maintenance of CNS immune tolerance in health as well as in the pathogenesis of MS. However, whether DCs are present in the normal CNS, as they are in so many, if not all, other tissues, remains hotly debated (see below) [80–83]. Regardless of how this resolves, it is clear that CNS antigens are present in, and capable of draining to, the cervical lymph nodes of MS patients and normal subjects [84]. Finally, most recently, it has been shown that depletion of DCs enhances EAE disease, confirming a role for DCs in braking autoimmune responses [51].

## 5. Experimental Autoimmune Encephalomyelitis (EAE): A Model of MS

Animal models play a key role in developing both an improved understanding of the pathogenesis of autoinflammatory diseases and disease related therapeutics [17,85,86]. In the case of MS, most effective treatments have been discovered using the EAE model and/or have beneficial effects in it [87]. However, some treatments have ameliorating effects in EAE that have not generalized to MS, in some cases leading to worsening disease indices [88]. While these findings represent a well-recognized limitation of the EAE model, many of the immunological mechanisms first identified in EAE are directly applicable to MS [87,88]. An important recent example of this is the discovery of a subgroup of T-helper-cells called Th17 cells, so-named because of their production of IL-17 [89]. This cell subset that plays an important role in disease pathogenesis, was defined in the EAE model [89] and subsequently found to have a significant role in MS [90–92]. These data are consistent with the long-held view that EAE is a T-cell mediated disease. However, it is clear that T-cells are not the only immune cell type involved in MS and EAE disease pathogenesis.

DC is intimately involved in T-cell function. Selected disruption of the antigen presenting capacity of DCs renders mice completely resistant to neuroinflammation, even after transferring disease-inducing T-cells, at least in part because functional DCs are required to allow the entry of pathogenic T-cells into the CNS [93]. Further characterization of DCs throughout the course of EAE have indicated that different subsets of DCs serve distinct functions: For example myeloid (conventional) DCs are involved in disease development, whilst plasmacytoid DCs, which produce interferons, are important in the development of T-regulatory cells and disease resolution [94–97]. Interestingly, it is this latter set of DCs that is absent in the IRF8 gene knockout mice considered in the previous section. These findings suggest a role for DCs in down-regulating immune responses in EAE and MS, and perhaps also a role in the maintenance of immune privilege of the CNS. Consistent with this view, DCs directly down regulate peripheral anti-CNS immune responses in inducing antigen specific T-regulatory cells in EAE [17,94]. However, a recent study utilizing a mouse model transgenic for a simian diphtheria toxin receptor expressed under the control of CD11c promoter, showed less activity for DCs in the EAE [98]. This finding highlights the ability of different phenotypes of DCs to prime T-cells and therefore to shape the disease causing immune response [99]. While these mechanisms are active in disease, it is not clear whether and how they may be operative in the healthy CNS.

## 6. Can DCs Be Found in the Normal CNS?

The concept of the “immune privilege” of the CNS has been with us since 1921 when Shirai first showed that heterologous tissue could be transplanted into the CNS and escape rejection [100]. Murphy and Sturm confirmed these results in 1923 [101] and later Tansley in 1946 [102]. However, Morton, in 1929, could not replicate these findings with human tumors, presumably because there was significant exposure of tumor to the meninges and/or cerebral ventricles, a factor noted by Sturm as potentially leading to tumor rejection [103]. These results suggested that the CNS parenchyma was ruled by a different immune paradigm than the meninges and ventricles. Peter Medawar used this heterologous tumor transplant model to demonstrate tolerance and the fact that it could be broken by peripheral immunization [104], a discovery that lead to a shared 1960 Nobel Prize for Medicine with MacFarlane Burnet. This finding suggests either that mechanisms in the immune system outside the CNS prevent peripheral immune activation and trafficking of inflammatory cells to the CNS to effect tumor rejection, or that inflammatory cells are simply excluded from accessing the CNS. Indeed, for many years these findings were considered to be due to the blood brain barrier (BBB), the lack of “competent” immune cells in the CNS, and formal lymphatic drainage, all thought to shield the CNS from systemic immune responses [3]. However, as methodologies have improved over the last 90 years, competent immune cells have been found in the CNS and more recently pathogenic T-cells were found to directly penetrate the intact BBB in some circumstances [92], probably abetted by DCs [82]. Further, competent immune cells (T-cells and DCs) “drain” from the CNS to the cervical lymph nodes, even in the absence of a formal lymphatic circulation [105]. However, unlike T-cells, the presence of dendritic cells in the healthy CNS is still currently controversial [80–83].

In 1988, Hickey described bone marrow-derived, fully competent antigen-presenting cells that express MHC Class II antigen located in the perivascular space of the cerebral vasculature, outside the BBB [106]. However, Hart and Fabre, previously found that there were no MHC Class II-positive DCs in the CNS parenchyma, but noted their presence in the meninges and choroid plexus [107]. These conflicting findings highlight the role of changing methodologies, their limitations and their influence on interpreting the presence or absence of cell subsets. Soon afterwards, there was a hint that antigen-presenting cells might exist in the brain parenchyma of the normal rat. However, Craggs and Webster indicated, with a personal communication from Hans Lassmann, that they might correspond to pericytes [108]. Later, Matyszak and Perry, found that there were very occasional DCs in the CNS parenchyma [109–111], the nature of which was not clear. Whilst attempts to identify DCs in the normal CNS parenchyma have been largely negative until recently, it is hard to completely exclude their presence, either in very small numbers or in highly localized areas. Indeed, recently Bulloch *et al*. and Prodinger *et al*. provided evidence that cells with a similar phenotype as peripheral DCs do develop in the normal CNS [82,83]. This finding is in agreement with the findings of Anandasabapathy *et al*. who showed that DCs expressing IRF8 developed from local CNS precursors, some, but not all, of which were bone marrow-derived and present in the parenchyma [112]. While the weight of emerging evidence supports the existence of DCs in the normal CNS, including the parenchyma, yet to be addressed is the critical question of whether they are capable of exiting the CNS and participating in systemic immune responses.

## 7. Could DCs Control Anti-CNS Immunity in the Periphery?

While highly autoreactive T-cells are deleted in the process of thymic selection, there are CNS-autoreactive T-cells in the circulation of many normal humans [42]. This suggests an active mechanism in which CNS avoids autoinflammation. This is best exemplified by the virtual lack of CNS-wide inflammation in the 2D2 transgenic mouse, when on the C57BL/6 background. The 2D2 mice have been engineered to transgenically express a MOG specific T-cell antigen receptor, such that between 50% and 90% of T-cells react to the MOG 35–55 peptide [41,113]. However, while 30% of these mice exhibit some optic nerve involvement, they rarely develop overt signs of CNS autoimmunity [41,113]. This tolerogenic mechanism may involve either IL-10 secreted from tolerogenic DCs, Tregs or Th2 cells with immunosuppressive secreted cytokines such as IL-4, IL-10, IL-13 and IL-9 [44]. In contrast, a similar mouse on the SJL genetic background, when engineered to express a TCR reactive to the MOG 92–106 peptide, did develop spontaneous CNS-wide inflammation with an incidence of up to 70% [40].

An important function of the immune system is to provide protection from disease and resolve inflammatory events without sustaining excessive damage. The disruption of this balance leads to either autoimmune-mediated tissue damage and/or increased susceptibility to infection. Additionally, in health, appropriate immune activation might also be responsible for the maintenance of CNS integrity [114–117]. Perhaps the most convincing evidence that this is the case is the recent finding that in MS, Alemtuzumab treatment was capable of reversing disability by, at least in part, increasing the number of autoreactive T-cell subsets that produce neurotrophic factors such as brain derived neurotrophic factor (BDNF) [118]. These bone fide CNS autoreactive T-cells were likely to have been activated by DCs, and it is well known that Alemtuzumab is capable of influencing DC function, particularly by increasing IRF8-mediated DC differentiation [119,120].

Modification of DC function in the periphery is also able to modify autoimmune neuroinflammation. We have shown that modification of DCs to produce and secrete increased amounts of IL-10 increases T-regulatory cell development [17]. T-regulatory development is also promoted by peripheral DCs expressing programmed cell death-1 (PD1) [51]. Thus DCs not only activate T-cells and support their proliferation, but also modify their development by expressing surface molecules and secreting cytokines. In keeping with this, a number of these DC-derived cytokines and surface molecules are associated with an increased susceptibility to MS. These observations raise the question as to where these potential immunoregulatory processes might take place [4,5,121].

## 8. The Cervical Lymph Node as a Site of CNS Immune Regulation

The cervical lymph nodes have been identified as peripheral sites where anti-CNS immune responses might be initiated and regulated [84,122]. The few DCs that are found in the CSF can traffic through the cribriform plate to the cervical lymph nodes, despite the lack of formal lymphatic drainage, (reviewed in [80,105]). Additionally, CNS antigens readily accumulate in the cervical lymph nodes [123]. Further, when labeled DCs are injected into the brain parenchyma, they are able to travel to cervical lymph nodes and elicit an immune response [124]. Indeed, in an elegant study, the immune response to a foreign antigen injected in the brain was shown to be initiated in the cervical lymph node, with activated T-cells then traveling to the CNS [122]. These data indicate that the cervical lymph nodes are a key site for the induction of anti-CNS immune responses. Consistent with a role in regulation of anti-CNS immune responses, there are also data from EAE mice to indicate that the cervical lymph nodes are also capable of dampening anti-CNS immunity [125]. Together, these findings indicate that CNS antigen, and perhaps DCs, travel to the cervical lymph nodes where they are involved in the regulation of anti-CNS immunity. This raises the possibility that the CNS can direct immune cells to the peripheral immune system and influence systemic anti-CNS immunity at the level of the cervical lymph nodes.

## 9. Targeted Therapy to the Cervical Lymph Nodes?

Most, if not all, MS therapeutics are delivered systemically. However, the recent findings implicating the cervical lymph nodes as significant regulators of anti-CNS immune responses raises the question as to whether benefit may be found in more discretely targeted therapies. The cervical lymph nodes could be accessed preferentially by delivering cytokines intra-nasally, via the lymphatics of the scalp or by direct infusion [105,126]. Using such approaches, it would seem feasible to deliver relatively small amounts of a formulation to attain therapeutic concentrations in the cervical lymph nodes, which might be expected to exhibit biological activity with a lesser propensity for systemic side effects. The potential for using less of what are often very expensive compounds to achieve the desired therapeutic effect, could also significantly reduce treatment costs.

Additionally, previously trialed compounds that have been found to be ineffective or associated with adverse side effects when delivered systemically, might find applicability when delivered locally. An example of this may be seen in the trials of systemic IL-10, which have been largely disappointing, perhaps because of rapid systemic clearance and consequently low local concentrations [127]. As some DCs favorably modulate anti-CNS immune responses by regulating their IL-10 secretion [17], localized, targeted delivery of IL-10 might achieve therapeutic concentrations in the cervical lymph nodes to modify DC-mediated T-cell activation.

## 10. Conclusions

The available data strongly suggest that DCs are involved in the regulation of anti-CNS immunity under normal conditions and in the development of autoimmune neuroinflammation. DCs appear to perform this task, at least in part, in the cervical lymph nodes. While these data raise many questions, which are yet to be satisfactorily answered, particularly the controversial issue of whether there exist functional DCs in the normal CNS parenchyma, they do have practical implications. Prime among these is the possibility of directing therapy to target DC-T-cell interactions focally, to the cervical lymph nodes, in order to enhance therapeutic efficacy and minimize side-effects, while reducing the economic burden associated with the treatment of neuroinflammatory disease.

## References

[1] Lassmann H., van Horssen J., Mahad D. (2012). Progressive multiple sclerosis: Pathology and pathogenesis. Nat. Rev. Neurol.

[2] Antel J., Antel S., Caramanos Z., Arnold D.L., Kuhlmann T. (2012). Primary progressive multiple sclerosis: Part of the MS disease spectrum or separate disease entity?. Acta Neuropathol.

[3] Goverman J. (2009). Autoimmune T cell responses in the central nervous system. Nat. Rev. Immunol.

[4] Patsopoulos N.A., Esposito F., Reischl J., Lehr S., Bauer D., Heubach J., Sandbrink R., Pohl C., Edan G., Kappos L. (2011). Genome-wide meta-analysis identifies novel multiple sclerosis susceptibility loci. Ann. Neurol.

[5] Sawcer S., Hellenthal G., Pirinen M., Spencer C.C., Patsopoulos N.A., Moutsianas L., Dilthey A., Su Z., Freeman C., Hunt S.E. (2011). Genetic risk and a primary role for cell-mediated immune mechanisms in multiple sclerosis. Nature.

[6] Bergamaschi L., Leone M.A., Fasano M.E., Guerini F.R., Ferrante D., Bolognesi E., Barizzone N., Corrado L., Naldi P., Agliardi C. (2010). HLA-class I markers and multiple sclerosis susceptibility in the Italian population. Genes Immun.

[7] D’Alfonso S., Bolognesi E., Guerini F.R., Barizzone N., Bocca S., Ferrante D., Castelli L., Bergamaschi L., Agliardi C., Ferrante P. (2008). A sequence variation in the MOG gene is involved in multiple sclerosis susceptibility in Italy. Genes Immun.

[8] Greer J.M., Pender M.P. (2005). The presence of glutamic acid at positions 71 or 74 in pocket 4 of the HLA-DRbeta1 chain is associated with the clinical course of multiple sclerosis. J. Neurol. Neurosurg. Psychiatry.

[9] Hafler D.A., Compston A., Sawcer S., Lander E.S., Daly M.J., de Jager P.L., de Bakker P.I., Gabriel S.B., Mirel D.B., Ivinson A.J. (2007). Risk alleles for multiple sclerosis identified by a genomewide study. N. Engl. J. Med.

[10] Raddassi K., Kent S.C., Yang J., Bourcier K., Bradshaw E.M., Seyfert-Margolis V., Nepom G.T., Kwok W.W., Hafler D.A. (2011). Increased frequencies of myelin oligodendrocyte glycoprotein/MHC class II-binding CD4 cells in patients with multiple sclerosis. J. Immunol.

[11] Ghoreschi K., Bruck J., Kellerer C., Deng C., Peng H., Rothfuss O., Hussain R.Z., Gocke A.R., Respa A., Glocova I. (2011). Fumarates improve psoriasis and multiple sclerosis by inducing type II dendritic cells. J. Exp. Med.

[12] Killestein J., Rudick R.A., Polman C.H. (2011). Oral treatment for multiple sclerosis. Lancet Neurol.

[13] Butovsky O., Koronyo-Hamaoui M., Kunis G., Ophir E., Landa G., Cohen H., Schwartz M. (2006). Glatiramer acetate fights against Alzheimer’s disease by inducing dendritic-like microglia expressing insulin-like growth factor 1. Proc. Natl. Acad. Sci. USA.

[14] Vieira P.L., Heystek H.C., Wormmeester J., Wierenga E.A., Kapsenberg M.L. (2003). Glatiramer acetate (copolymer-1, copaxone) promotes Th2 cell development and increased IL-10 production through modulation of dendritic cells. J. Immunol.

[15] Aung L.L., Fitzgerald-Bocarsly P., Dhib-Jalbut S., Balashov K. (2010). Plasmacytoid dendritic cells in multiple sclerosis: Chemokine and chemokine receptor modulation by interferon-beta. J. Neuroimmunol.

[16] Lande R., Gafa V., Serafini B., Giacomini E., Visconti A., Remoli M.E., Severa M., Parmentier M., Ristori G., Salvetti M. (2008). Plasmacytoid dendritic cells in multiple sclerosis: intracerebral recruitment and impaired maturation in response to interferon-beta. J. Neuropathol. Exp. Neurol.

[17] Tsai V.W., Mohammad M.G., Tolhurst O., Breit S.N., Sawchenko P.E., Brown D.A. (2011). CCAAT/enhancer binding protein-delta expression by dendritic cells regulates CNS autoimmune inflammatory disease. J. Neurosci.

[18] Ponomarev E.D., Dittel B.N. (2005). Gamma delta T cells regulate the extent and duration of inflammation in the central nervous system by a Fas ligand-dependent mechanism. J. Immunol.

[19] Merelli E., Sola P., Faglioni P., Giordani S., Mussini D., Montagnani G. (1991). Natural killer cells and lymphocyte subsets in active MS and acute inflammation of the CNS. Acta Neurol. Scand.

[20] Sun D., Meyermann R., Wekerle H. (1988). Cytotoxic T cells in autoimmune disease of the central nervous system. Ann. N. Y. Acad. Sci.

[21] Raedler A., Bredow G., Kirch W., Thiele H.G., Greten H. (1986). *In vivo* activated peripheral T cells in autoimmune disease. J. Clin. Lab. Immunol.

[22] Jacobson S., Flerlage M.L., McFarland H.F. (1985). Impaired measles virus-specific cytotoxic T cell responses in multiple sclerosis. J. Exp. Med.

[23] Hafler D.A., Buchsbaum M., Johnson D., Weiner H.L. (1985). Phenotypic and functional analysis of T cells cloned directly from the blood and cerebrospinal fluid of patients with multiple sclerosis. Ann. Neurol.

[24] Colombo M., Dono M., Gazzola P., Roncella S., Valetto A., Chiorazzi N., Mancardi G.L., Ferrarini M. (2000). Accumulation of clonally related B lymphocytes in the cerebrospinal fluid of multiple sclerosis patients. J. Immunol.

[25] Terasaki P.I., Park M.S., Opelz G., Ting A. (1976). Multiple sclerosis and high incidence of a B lymphocyte antigen. Science.

[26] Compston D.A., Batchelor J.R., McDonald W.I. (1976). B-lymphocyte alloantigens associated with multiple sclerosis. Lancet.

[27] Vermersch P., Benrabah R., Schmidt N., Zephir H., Clavelou P., Vongsouthi C., Dubreuil P., Moussy A., Hermine O. (2012). Masitinib treatment in patients with progressive multiple sclerosis: A randomized pilot study. BMC Neurol.

[28] Sayed B.A., Walker M.E., Brown M.A. (2011). Cutting edge: Mast cells regulate disease severity in a relapsing-remitting model of multiple sclerosis. J. Immunol.

[29] Olsson Y. (1974). Mast cells in plaques of multiple sclerosis. Acta Neurol. Scand.

[30] Barnett M.H., Henderson A.P., Prineas J.W. (2006). The macrophage in MS: Just a scavenger after all? Pathology and pathogenesis of the acute MS lesion. Mult. Scler.

[31] Doring A., Yong V.W. (2011). The good, the bad and the ugly. Macrophages/microglia with a focus on myelin repair. Front. Biosci.

[32] Almolda B., Gonzalez B., Castellano B. (2011). Antigen presentation in EAE: Role of microglia, macrophages and dendritic cells. Front. Biosci.

[33] Kort J.J., Kawamura K., Fugger L., Weissert R., Forsthuber T.G. (2006). Efficient presentation of myelin oligodendrocyte glycoprotein peptides but not protein by astrocytes from HLA-DR2 and HLA-DR4 transgenic mice. J. Neuroimmunol.

[34] Constantinescu C.S., Tani M., Ransohoff R.M., Wysocka M., Hilliard B., Fujioka T., Murphy S., Tighe P.J., Das Sarma J., Trinchieri G., Rostami A. (2005). Astrocytes as antigen-presenting cells: Expression of IL-12/IL-23. J. Neurochem.

[35] Zeinstra E., Wilczak N., De Keyser J. (2003). Reactive astrocytes in chronic active lesions of multiple sclerosis express co-stimulatory molecules B7-1 and B7-2. J. Neuroimmunol.

[36] Tan L., Gordon K.B., Mueller J.P., Matis L.A., Miller S.D. (1998). Presentation of proteolipid protein epitopes and B7-1-dependent activation of encephalitogenic T cells by IFN-gamma-activated SJL/J astrocytes. J. Immunol.

[37] Traugott U., Lebon P. (1988). Interferon-γ and Ia antigen are present on astrocytes in active chronic multiple sclerosis lesions. J. Neurol. Sci.

[38] Traugott U., Raine C.S. (1985). Multiple sclerosis. Evidence for antigen presentation *in situ* by endothelial cells and astrocytes. J. Neurol. Sci.

[39] Fontana A., Fierz W., Wekerle H. (1984). Astrocytes present myelin basic protein to encephalitogenic T-cell lines. Nature.

[40] Pollinger B., Krishnamoorthy G., Berer K., Lassmann H., Bosl M.R., Dunn R., Domingues H.S., Holz A., Kurschus F.C., Wekerle H. (2009). Spontaneous relapsing-remitting EAE in the SJL/J mouse: MOG-reactive transgenic T cells recruit endogenous MOG-specific B cells. J. Exp. Med.

[41] Bettelli E., Pagany M., Weiner H.L., Linington C., Sobel R.A., Kuchroo V.K. (2003). Myelin oligodendrocyte glycoprotein-specific T cell receptor transgenic mice develop spontaneous autoimmune optic neuritis. J. Exp. Med.

[42] Crawford M.P., Yan S.X., Ortega S.B., Mehta R.S., Hewitt R.E., Price D.A., Stastny P., Douek D.C., Koup R.A., Racke M.K., Karandikar N.J. (2004). High prevalence of autoreactive, neuroantigen-specific CD8^+^ T cells in multiple sclerosis revealed by novel flow cytometric assay. Blood.

[43] Sapoznikov A., Jung S. (2008). Probing *in vivo* dendritic cell functions by conditional cell ablation. Immunol. Cell Biol.

[44] Colton C.A. (2012). Immune heterogeneity in neuroinflammation: Dendritic cells in the brain. J. Neuroimmune Pharmacol..

[45] Sellebjerg F., Hesse D., Limborg S., Lund H., Sondergaard H., Krakauer M., Sorensen P. (2012). Dendritic cell, monocyte and T cell activation and response to glatiramer acetate in multiple sclerosis. Mult. Scler..

[46] Galicia-Rosas G., Pikor N., Schwartz J.A., Rojas O., Jian A., Summers-Deluca L., Ostrowski M., Nuesslein-Hildesheim B., Gommerman J.L. (2012). A Sphingosine-1-phosphate receptor 1-directed agonist reduces central nervous system inflammation in a plasmacytoid dendritic cell-dependent manner. J. Immunol.

[47] Peng H., Guerau-de-Arellano M., Mehta V.B., Yang Y., Huss D.J., Papenfuss T.L., Lovett-Racke A.E., Racke M.K. (2012). Dimethyl fumarate inhibits dendritic cell maturation via nuclear factor κB (NF-κB) and extracellular signal-regulated kinase 1 and 2 (ERK1/2) and mitogen stress-activated kinase 1 (MSK1) signaling. J. Biol. Chem.

[48] Kong W., Yen J.H., Ganea D. (2011). Docosahexaenoic acid prevents dendritic cell maturation, inhibits antigen-specific Th1/Th17 differentiation and suppresses experimental autoimmune encephalomyelitis. Brain Behav. Immun.

[49] Pluchino S., Zanotti L., Brambilla E., Rovere-Querini P., Capobianco A., Alfaro-Cervello C., Salani G., Cossetti C., Borsellino G., Battistini L. (2009). Immune regulatory neural stem/precursor cells protect from central nervous system autoimmunity by restraining dendritic cell function. PLoS One.

[50] Martin-Fontecha A., Lanzavecchia A., Sallusto F. (2009). Dendritic cell migration to peripheral lymph nodes. Handb. Exp. Pharmacol..

[51] Yogev N., Frommer F., Lukas D., Kautz-Neu K., Karram K., Ielo D., von Stebut E., Probst H.C., van den Broek M., Riethmacher D. (2012). Dendritic cells ameliorate Aautoimmunity in the CNS by controlling the homeostasis of PD-1 Receptor(+) regulatory T Cells. Immunity.

[52] McFarland H.F., Greenstein J., McFarlin D.E., Eldridge R., Xu X.H., Krebs H. (1984). Family and twin studies in multiple sclerosis. Ann. N. Y. Acad. Sci.

[53] Simon K.C., Munger K.L., Ascherio A. (2012). Vitamin D and multiple sclerosis: Epidemiology, immunology, and genetics. Curr. Opin. Neurol.

[54] Ascherio A., Munger K.L., Giovannucci E. (2011). Sun exposure and vitamin D are independent risk factors for CNS demyelination. Neurology.

[55] Munger K.L., Ascherio A. (2011). Prevention and treatment of MS: Studying the effects of vitamin D. Mult. Scler.

[56] Hart P.H., Gorman S., Finlay-Jones J.J. (2011). Modulation of the immune system by UV radiation: More than just the effects of vitamin D?. Nat. Rev. Immunol.

[57] Kushwah R., Hu J. (2011). Role of dendritic cells in the induction of regulatory T cells. Cell Biosci.

[58] Schwab N., Zozulya A.L., Kieseier B.C., Toyka K.V., Wiendl H. (2010). An imbalance of two functionally and phenotypically different subsets of plasmacytoid dendritic cells characterizes the dysfunctional immune regulation in multiple sclerosis. J. Immunol.

[59] Ilarregui J.M., Rabinovich G.A. (2010). Tolerogenic dendritic cells in the control of autoimmune neuroinflammation: An emerging role of protein-glycan interactions. Neuroimmunomodulation.

[60] Nemeth E., Baird A.W., O’Farrelly C. (2009). Microanatomy of the liver immune system. Semin. Immunopathol.

[61] Lehner T. (2008). Special regulatory T cell review: The resurgence of the concept of contrasuppression in immunoregulation. Immunology.

[62] Trucco M., Giannoukakis N. (2007). Immunoregulatory dendritic cells to prevent and reverse new-onset type 1 diabetes mellitus. Expert Opin. Biol. Ther.

[63] Lutz M.B., Kurts C. (2009). Induction of peripheral CD4^+^ T-cell tolerance and CD8^+^ T-cell cross-tolerance by dendritic cells. Eur. J. Immunol.

[64] Bonasio R., Scimone M.L., Schaerli P., Grabie N., Lichtman A.H., von Andrian U.H. (2006). Clonal deletion of thymocytes by circulating dendritic cells homing to the thymus. Nat. Immunol.

[65] Luckey U., Schmidt T., Pfender N., Romer M., Lorenz N., Martin S.F., Bopp T., Schmitt E., Nikolaev A., Yogev N. (2012). Crosstalk of regulatory T cells and tolerogenic dendritic cells prevents contact allergy in subjects with low zone tolerance. J. Allergy Clin. Immunol.

[66] Moreau A., Varey E., Beriou G., Hill M., Bouchet-Delbos L., Segovia M., Cuturi M.C. (2012). Tolerogenic dendritic cells and negative vaccination in transplantation: From rodents to clinical trials. Front. Immunol.

[67] Amodio G., Gregori S. (2012). Dendritic cells a double-edge sword in autoimmune responses. Front. Immunol.

[68] Monti P., Leone B.E., Zerbi A., Balzano G., Cainarca S., Sordi V., Pontillo M., Mercalli A., di Carlo V., Allavena P., Piemonti L. (2004). Tumor-derived MUC1 mucins interact with differentiating monocytes and induce IL-10highIL-12low regulatory dendritic cell. J. Immunol.

[69] Janikashvili N., Bonnotte B., Katsanis E., Larmonier N. (2011). The dendritic cell-regulatory T lymphocyte crosstalk contributes to tumor-induced tolerance. Clin. Dev. Immunol.

[70] Thomson A.W., Knolle P.A. (2010). Antigen-presenting cell function in the tolerogenic liver environment. Nat. Rev. Immunol.

[71] Sumpter T.L., Lunz J.G.r., Castellaneta A., Matta B., Tokita D., Turnquist H.R., Mazariegos G.V., Demetris A.J., Thomson A.W. (2009). Dendritic cell immunobiology in relation to liver transplant outcome. Front. Biosci.

[72] Benseler V., McCaughan G.W., Schlitt H.J., Bishop G.A., Bowen D.G., Bertolino P. (2007). The liver: A special case in transplantation tolerance. Semin. Liver Dis.

[73] Hambleton S., Salem S., Bustamante J., Bigley V., Boisson-Dupuis S., Azevedo J., Fortin A., Haniffa M., Ceron-Gutierrez L., Bacon C.M. (2011). IRF8 mutations and human dendritic-cell immunodeficiency. N. Engl. J. Med.

[74] Lee W.I., Huang J.L., Yeh K.W., Jaing T.H., Lin T.Y., Huang Y.C., Chiu C.H. (2011). Immune defects in active mycobacterial diseases in patients with primary immunodeficiency diseases (PIDs). J. Formos. Med. Assoc.

[75] Doring Y., Soehnlein O., Drechsler M., Shagdarsuren E., Chaudhari S.M., Meiler S., Hartwig H., Hristov M., Koenen R.R., Hieronymus T. (2012). Hematopoietic interferon regulatory factor 8-deficiency accelerates atherosclerosis in mice. Arterioscler. Thromb. Vasc. Biol.

[76] Tailor P., Tamura T., Morse H.C., Ozato K. (2008). The BXH2 mutation in IRF8 differentially impairs dendritic cell subset development in the mouse. Blood.

[77] Tailor P., Tamura T., Kong H.J., Kubota T., Kubota M., Borghi P., Gabriele L., Ozato K. (2007). The feedback phase of type I interferon induction in dendritic cells requires interferon regulatory factor 8. Immunity.

[78] The International Multiple Sclerosis Genetics Consortium (2011). The genetic association of variants in CD6, TNFRSF1A and IRF8 to multiple sclerosis: A multicenter case-control study. PLoS One.

[79] De Jager P.L., Jia X., Wang J., de Bakker P.I., Ottoboni L., Aggarwal N.T., Piccio L., Raychaudhuri S., Tran D., Aubin C. (2009). Meta-analysis of genome scans and replication identify CD6, IRF8 and TNFRSF1A as new multiple sclerosis susceptibility loci. Nat. Genet.

[80] Ransohoff R.M., Brown M.A. (2012). Innate immunity in the central nervous system. J. Clin. Invest.

[81] Ransohoff R.M., Perry V.H. (2009). Microglial physiology: Unique stimuli, specialized responses. Annu. Rev. Immunol.

[82] Prodinger C., Bunse J., Kruger M., Schiefenhovel F., Brandt C., Laman J.D., Greter M., Immig K., Heppner F., Becher B., Bechmann I. (2011). CD11c-expressing cells reside in the juxtavascular parenchyma and extend processes into the glia limitans of the mouse nervous system. Acta Neuropathol.

[83] Bulloch K., Miller M.M., Gal-Toth J., Milner T.A., Gottfried-Blackmore A., Waters E.M., Kaunzner U.W., Liu K., Lindquist R., Nussenzweig M.C. (2008). CD11c/EYFP transgene illuminates a discrete network of dendritic cells within the embryonic, neonatal, adult, and injured mouse brain. J. Comp. Neurol.

[84] Van Zwam M., Huizinga R., Melief M.J., Wierenga-Wolf A.F., van Meurs M., Voerman J.S., Biber K.P., Boddeke H.W., Hopken U.E., Meisel C. (2009). Brain antigens in functionally distinct antigen-presenting cell populations in cervical lymph nodes in MS and EAE. J. Mol. Med.

[85] Brown D.A., Sawchenko P.E. (2007). Time course and distribution of inflammatory and neurodegenerative events suggest structural bases for the pathogenesis of experimental autoimmune encephalomyelitis. J. Comp. Neurol.

[86] Lucchinetti C.F., Popescu B.F., Bunyan R.F., Moll N.M., Roemer S.F., Lassmann H., Bruck W., Parisi J.E., Scheithauer B.W., Giannini C. (2011). Inflammatory cortical demyelination in early multiple sclerosis. N. Engl. J. Med.

[87] Steinman L., Zamvil S.S. (2006). How to successfully apply animal studies in experimental allergic encephalomyelitis to research on multiple sclerosis. Ann. Neurol.

[88] Slavin A., Kelly-Modis L., Labadia M., Ryan K., Brown M.L. (2010). Pathogenic mechanisms and experimental models of multiple sclerosis. Autoimmunity.

[89] Langrish C.L., Chen Y., Blumenschein W.M., Mattson J., Basham B., Sedgwick J.D., McClanahan T., Kastelein R.A., Cua D.J. (2005). IL-23 drives a pathogenic T cell population that induces autoimmune inflammation. J. Exp. Med.

[90] Lovett-Racke A.E., Yang Y., Racke M.K. (2011). Th1 versus Th17: Are T cell cytokines relevant in multiple sclerosis?. Biochim Biophys. Acta.

[91] Fletcher J.M., Lalor S.J., Sweeney C.M., Tubridy N., Mills K.H. (2010). T cells in multiple sclerosis and experimental autoimmune encephalomyelitis. Clin. Exp. Immunol.

[92] Kebir H., Kreymborg K., Ifergan I., Dodelet-Devillers A., Cayrol R., Bernard M., Giuliani F., Arbour N., Becher B., Prat A. (2007). Human TH17 lymphocytes promote blood-brain barrier disruption and central nervous system inflammation. Nat. Med.

[93] Greter M., Heppner F.L., Lemos M.P., Odermatt B.M., Goebels N., Laufer T., Noelle R.J., Becher B. (2005). Dendritic cells permit immune invasion of the CNS in an animal model of multiple sclerosis. Nat. Med.

[94] Bailey-Bucktrout S.L., Caulkins S.C., Goings G., Fischer J.A., Dzionek A., Miller S.D. (2008). Cutting edge: Central nervous system plasmacytoid dendritic cells regulate the severity of relapsing experimental autoimmune encephalomyelitis. J. Immunol.

[95] Bailey S.L., Schreiner B., McMahon E.J., Miller S.D. (2007). CNS myeloid DCs presenting endogenous myelin peptides “preferentially” polarize CD4^+^ T(H)-17 cells in relapsing EAE. Nat. Immunol.

[96] Miller S.D., McMahon E.J., Schreiner B., Bailey S.L. (2007). Antigen presentation in the CNS by myeloid dendritic cells drives progression of relapsing experimental autoimmune encephalomyelitis. Ann. N. Y. Acad. Sci.

[97] Bailey S.L., Carpentier P.A., McMahon E.J., Begolka W.S., Miller S.D. (2006). Innate and adaptive immune responses of the central nervous system. Crit. Rev. Immunol.

[98] Isaksson M., Lundgren B.A., Ahlgren K.M., Kampe O., Lobell A. (2012). Conditional DC depletion does not affect priming of encephalitogenic Th cells in EAE. Eur. J. Immunol.

[99] Becher B., Greter M. (2012). Acquitting an APC: DCs found “not guilty” after trial by ablation. Eur. J. Immunol.

[100] Shirai Y. (1921). On the transplantation ofthe rat sarcoma in adult heterogeneous animals. Jpn. Med. World.

[101] Murphy J.B., Sturm E. (1923). Conditions determining the transplantability of tissues in the brain. J. Exp. Med.

[102] Tansley K. (1946). The development of the rat eye in graft. J. Exp. Biol.

[103] Morton J.J. (1929). On the failure in hetroplastic transplantation of human maamary carcinoma into the brains of rats. J. Cancer Res..

[104] Billingham R.E., Brent L., Medawar P.B. (1953). Actively acquired tolerance of foreign cells. Nature.

[105] Weller R.O., Galea I., Carare R.O., Minagar A. (2009). Pathophysiology of the lymphatic drainage of the central nervous system: Implications for pathogenesis and therapy of multiple sclerosis. Pathophysiology.

[106] Hickey W.F., Kimura H. (1988). Perivascular microglial cells of the CNS are bone marrow-derived and present antigen *in vivo*. Science.

[107] Hart D.N., Fabre J.W. (1981). Demonstration and characterization of Ia-positive dendritic cells in the interstitial connective tissues of rat heart and other tissues, but not brain. J. Exp. Med.

[108] Craggs R.I., Webster H.D. (1985). Ia antigens in the normal rat nervous system and in lesions of experimental allergic encephalomyelitis. Acta Neuropathol.

[109] Matyszak M.K., Perry V.H. (1996). The potential role of dendritic cells in immune-mediated inflammatory diseases in the central nervous system. Neuroscience.

[110] Matyszak M.K., Perry V.H. (1996). Delayed-type hypersensitivity lesions in the central nervous system are prevented by inhibitors of matrix metalloproteinases. J. Neuroimmunol.

[111] Matyszak M.K., Perry V.H. (1996). A comparison of leucocyte responses to heat-killed bacillus Calmette-Guerin in different CNS compartments. Neuropathol. Appl. Neurobiol.

[112] Anandasabapathy N., Victora G.D., Meredith M., Feder R., Dong B., Kluger C., Yao K., Dustin M.L., Nussenzweig M.C., Steinman R.M., Liu K. (2011). Flt3L controls the development of radiosensitive dendritic cells in the meninges and choroid plexus of the steady-state mouse brain. J. Exp. Med.

[113] Bettelli E., Baeten D., Jager A., Sobel R.A., Kuchroo V.K. (2006). Myelin oligodendrocyte glycoprotein-specific T and B cells cooperate to induce a Devic-like disease in mice. J. Clin. Invest.

[114] Schwartz M., Shechter R. (2010). Systemic inflammatory cells fight off neurodegenerative disease. Nat. Rev. Neurol.

[115] Shechter R., London A., Varol C., Raposo C., Cusimano M., Yovel G., Rolls A., Mack M., Pluchino S., Martino G., Jung S., Schwartz M. (2009). Infiltrating blood-derived macrophages are vital cells playing an anti-inflammatory role in recovery from spinal cord injury in mice. PLoS Med.

[116] Schwartz M., Ziv Y. (2008). Immunity to self and self-maintenance: A unified theory of brain pathologies. Trends Immunol.

[117] Moalem G., Leibowitz-Amit R., Yoles E., Mor F., Cohen I.R., Schwartz M. (1999). Autoimmune T cells protect neurons from secondary degeneration after central nervous system axotomy. Nat. Med.

[118] Jones J.L., Anderson J.M., Phuah C.L., Fox E.J., Selmaj K., Margolin D., Lake S.L., Palmer J., Thompson S.J., Wilkins A. (2010). Improvement in disability after alemtuzumab treatment of multiple sclerosis is associated with neuroprotective autoimmunity. Brain.

[119] Kirsch B.M., Haidinger M., Zeyda M., Bohmig G.A., Tombinsky J., Muhlbacher F., Watschinger B., Horl W.H., Saemann M.D. (2006). Alemtuzumab (Campath-1H) induction therapy and dendritic cells: Impact on peripheral dendritic cell repertoire in renal allograft recipients. Transpl. Immunol.

[120] Cisse B., Caton M.L., Lehner M., Maeda T., Scheu S., Locksley R., Holmberg D., Zweier C., den Hollander N.S., Kant S.G. (2008). Transcription factor E2-2 is an essential and specific regulator of plasmacytoid dendritic cell development. Cell.

[121] Riveros C., Mellor D., Gandhi K.S., McKay F.C., Cox M.B., Berretta R., Vaezpour S.Y., Inostroza-Ponta M., Broadley S.A., Heard R.N. (2010). A transcription factor map as revealed by a genome-wide gene expression analysis of whole-blood mRNA transcriptome in multiple sclerosis. PLoS One.

[122] Ling C., Sandor M., Suresh M., Fabry Z. (2006). Traumatic injury and the presence of antigen differentially contribute to T-cell recruitment in the CNS. J. Neurosci.

[123] Ling C., Sandor M., Fabry Z. (2003). *In situ* processing and distribution of intracerebrally injected OVA in the CNS. J. Neuroimmunol.

[124] Karman J., Chu H.H., Co D.O., Seroogy C.M., Sandor M., Fabry Z. (2006). Dendritic cells amplify T cell-mediated immune responses in the central nervous system. J. Immunol.

[125] Mana P., Fordham S.A., Staykova M.A., Correcha M., Silva D., Willenborg D.O., Linares D. (2009). Demyelination caused by the copper chelator cuprizone halts T cell mediated autoimmune neuroinflammation. J. Neuroimmunol.

[126] Weller R.O., Engelhardt B., Phillips M.J. (1996). Lymphocyte targeting of the central nervous system: A review of afferent and efferent CNS-immune pathways. Brain Pathol.

[127] Herfarth H., Scholmerich J. (2002). IL-10 therapy in Crohn’s disease: At the crossroads. Treatment of Crohn’s disease with the anti-inflammatory cytokine interleukin 10. Gut.

